# Analysis and forecast of dengue incidence in urban Colombo, Sri Lanka

**DOI:** 10.1186/s12976-020-00134-7

**Published:** 2021-01-07

**Authors:** KKWH Erandi, SSN Perera, AC Mahasinghe

**Affiliations:** Research & Development Center for Mathematical Modelling, Department of Mathematics, University of Colombo, Colombo, 00003 Sri Lanka

**Keywords:** Dengue, IR model, Seasonal pattern, Fourier analysis

## Abstract

**Background:**

Understanding the dynamical behavior of dengue transmission is essential in designing control strategies. Mathematical models have become an important tool in describing the dynamics of a vector borne disease. Classical compartmental models are well–known method used to identify the dynamical behavior of spread of a vector borne disease. Due to use of fixed model parameters, the results of classical compartmental models do not match realistic nature. The aim of this study is to introduce time in varying model parameters, modify the classical compartmental model by improving its predictability power.

**Results:**

In this study, *per–capita vector density* has been chosen as the time in varying model parameter. The dengue incidences, rainfall and temperature data in urban Colombo are analyzed using Fourier mathematical analysis tool. Further, periodic pattern of the reported dengue incidences and meteorological data and correlation of dengue incidences with meteorological data are identified to determine climate data–driven *per–capita vector density* parameter function. By considering that the vector dynamics occurs in faster time scale compares to host dynamics, a two dimensional data–driven compartmental model is derived with aid of classical compartmental models. Moreover, a function for *per–capita vector density* is introduced to capture the seasonal pattern of the disease according to the effect of climate factors in urban Colombo.

**Conclusions:**

The two dimensional data–driven compartmental model can be used to predict weekly dengue incidences upto 4 weeks. Accuracy of the model is evaluated using relative error function and the model can be used to predict more than 75% accurate data.

## Introduction

Dengue is a mosquito–borne tropical viral disease that has rapidly spread during the past few decades and has become one of the major public health issues. Within the past five decades, the magnitude of reported number of incidents has increased by thirty fold and mainly reported from tropical and subtropical regions [1, 2]. According to World Health Organization (WHO), 0.4 to 1.3 million dengue cases are reported annually [2].

**Fig. 1:**
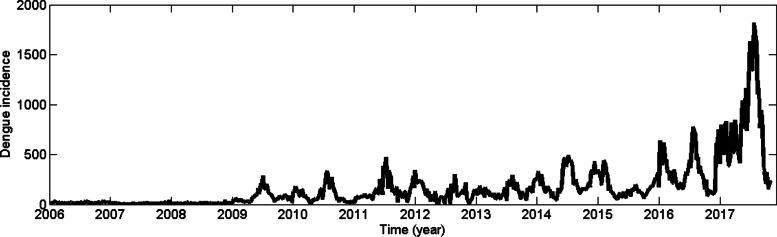
Reported weekly dengue incidence from 2006 January to 2017 December, Colombo Municipal Council area (Sources: Epidemiology Unit, Ministry of Health, Sri Lanka)

Sri Lanka is a tropical country which has been affected by dengue for over two decades and the infection has now gained the status of an endemic disease. Dengue was serologically confirmed in 1962 and the country experienced its first outbreak during 1965–1966 [3, 4]. Since then, several dengue outbreaks occurred and the worst was reported in 2017 with 186,101 cases [5, 6]. Approximately 25% of the dengue cases of the country were reported from the Colombo Municipal Council (CMC) area in every year [6]. CMC area is the most urbanized area in the country [7]. Figure 1 illustrates the trend of weekly dengue infection in CMC area from 2006 to 2017.

Though the first dengue vaccine was licensed in 2015, vaccine performance is dependent on serostatus [8]. That is, the efficacy of the vaccine is high and the vaccine is safe for those who have had a previous dengue infection (seropositive). Moreover, the vaccine increases the risk of developing severe dengue for seronegative vaccinees when they experience a natural dengue infection approximately 3 years after vaccination. Hence, still the main prevention strategy is vector control [2]. For countries with limited resources like Sri Lanka, it is necessary to identify the dynamics of the dengue spread thoroughly to determine more efficient control strategies. Accordingly, understanding the trend of spread of the disease is vital in prevention of dengue in Sri Lanka.

One of the key factor which affects the trend of spread of dengue is *per–capita vector density*, which is the number of adult mosquitoes per human. Considering a pragmatic situation, calculating *per capita–vector density* is an infeasible task, in particular when there is no method available to distinguish infected vectors from the uninfected. Having said that, there are certain measurable factors on which the *per–capita vector density* depends. For an example, air temperature effects water temperature at the breeding place of mosquitoes and consequently speedup the egg hatching process [9–11]. Moreover, vectors perform two–fold survival and produce more eggs under a proper combination of temperature and rainfall [11]. Further, a number of studies have been conducted to study the effects of meteorological factors on vector density in different countries including China, Taiwan, Indonesia and Brazil [12–17]. In addition, several previous works have examined the influence of weather–related parameters on dengue distribution in different regions of Sri Lanka [7, 18–22]. Accordingly, reported dengue incidents show a strong correlation with rainfall in Colombo with different time lags [21, 22]. Moreover, the disease occurs every year within or soon after monsoon seasons and the country is influenced by two monsoon seasons, Northeast monsoon season from December to February and Southwest monsoon season from May to September [23]. Further, average temperature of Sri Lanka varies from 17^∘^*C* to 35^∘^*C* which is ideal for dengue transmission. Motivated from all these, it is an essential task to quantify the impact of climate factors for spread of dengue disease in particular and *per–capita vector density* in general.

In order to capture the transmission pattern of the dengue virus which obviously correlates with the above–mentioned climate factors, it is natural to look into the well–known mathematical models of disease transmission based on the classical compartmental model. Most prominent is the model known as the SIR (susceptible, infected and recovered) compartmental model [24]. One of the main challenges with the derived SIR model for dengue transmission is estimating *per–capita vector density*. To overcome this problem, we design a simple data driven quasi–equilibrium IR model to capture the dynamical behavior of dengue transmission, which enables us to determine realistic control strategies. Further, we compare the dynamical behavior of infected host population in data driven quasi–equilibrium model with classical SIR model. Then we use reported dengue incidences and climate data in Colombo to determine climate dependent parameters in data driven quasi–equilibrium model. Finally, we discuss the accuracy of the model.

This paper is organized as follows. First we derive a two dimensional quasi–equilibrium IR model by adopting the classical SIR model as the basis in “Model development” section. Then we develop the data–driven compartmental model in “Development of a data-driven mathematical model” section. In the same section, we analyze the recorded climate data and reported dengue incidences. Consequently, we derive some results on the periodic pattern of dengue in urban Colombo. Then we present numerical results and discuss the accuracy of the model by defining the interval map in “Numerical results” section. Finally, we discuss our findings within the context of the literature in “Discussion” and conclude our remarks in “Conclusion” sections.

## Methodology

### Model development

#### Classical SIR model

The classical SIR model was introduced by Kermack and McKendrick [24], depending on the fact that any population can be divided into three compartments *susceptible*, *infected* and *recovered*, each containing individuals that are identical in terms of their status with respect to the disease. The classical SIR model can be modified to describe the interaction between susceptible human and infected vector populations using system of non–linear ordinary differential equations.

**Fig. 2:**
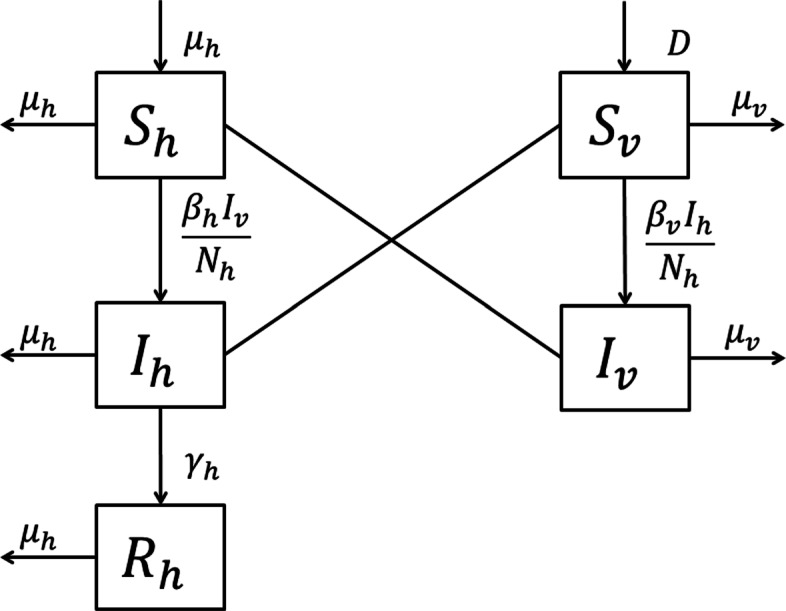
Schematic of the five compartments dengue transmission model. The arrows represent transitions between epidemiological classes, whereas the lines represent interactions between humans and vectors

In the modified model, the host population (*N*_*h*_) is divided into three compartments, susceptible humans (*S*_*h*_), infected humans (*I*_*h*_) and recovered humans (*R*_*h*_). Since vectors’ life cycle is 1–2 weeks and the infected period ends with their death, the vector population (*N*_*v*_) is divided into two compartments, susceptible vectors (*S*_*v*_) and infected vectors (*I*_*v*_). Moreover, the dynamics of the vectors occur at a faster rate compared to the host dynamics. Hence, we assume the host population (*N*_*h*_) and reproduction and mortality rate of the host (*μ*_*h*_) are constant. Further, we assume the vectors reproduce at a constant rate. Hence, the recruitment rate (*D*) and the mortality rate of vectors (*μ*_*v*_) are constant, while the total vector population too remains constant. Now, a schematic representation of the five compartment model is shown in Fig. 2 and the dynamical behavior of these five compartments can be explained as in system (1a) to (1e).

1a $$\begin{array}{*{20}l} \frac{dS_{h}}{dt} = \mu_{h}\left(N_{h} -S_{h}\right) - \frac{\beta_{h}}{N_{h}}I_{v} S_{h},  \end{array} $$

1b $$\begin{array}{*{20}l} \frac{dI_{h}}{dt} = \frac{\beta_{h}}{N_{h}}I_{v} S_{h} -(\mu_{h} + \gamma_{h})I_{h}, \end{array} $$

1c $$\begin{array}{*{20}l} \frac{dR_{h}}{dt} = \gamma_{h} I_{h} - \mu_{h} R_{h}, \end{array} $$

1d $$\begin{array}{*{20}l} \frac{dS_{v}}{dt} = D N_{v}-\mu_{v}S_{v} - \frac{\beta_{v}}{N_{h}}S_{v} I_{h}, \end{array} $$

1e $$\begin{array}{*{20}l} \frac{dI_{v}}{dt} = \frac{\beta_{v}}{N_{h}}S_{v} I_{h} -\mu_{v} I_{v}. \end{array} $$

with

2a $$\begin{array}{*{20}l} S_{h} + I_{h} + R_{h} = N_{h}, \end{array} $$

2b $$\begin{array}{*{20}l} S_{v} + I_{v} = N_{v}. \end{array} $$

The model assume all new–born are susceptible for both populations and populations are removed from each compartment due to natural death. The main assumption of this model is, dengue virus can be transmitted to susceptible human through infected vectors and transmitted to susceptible vectors through infected humans only. New infections occur as a result of contact between infected and susceptible individuals. Once the susceptible individuals become infected with the disease, they move to the relevant infected compartment. Once the infected hosts recovered from the disease, they move to the recovered compartment. Parameters *β*_*v*_, *β*_*h*_ and *γ*_*h*_ denote the transmission rate for host to vector, transmission rate for vector to host and host recovery rate, respectively.

We normalize the SIR model by taking $S = \frac {S_{h}}{N_{h}}$, $I = \frac {I_{h}}{N_{h}}$, $R = \frac {R_{h}}{N_{h}}$, $U = \frac {S_{v}}{N_{v}}$ and $V = \frac {I_{v}}{N_{v}}$. Then *S*+*I*+*R*=1 for host and *U*+*V*=1 for vector.

3a $$\begin{array}{*{20}l} \frac{dS}{dt} = \mu_{h}(1 -S) - \beta_{h} \frac{N_{v}}{N_{h}}V S, \end{array} $$

3b $$\begin{array}{*{20}l} \frac{dI}{dt} = \beta_{h} \frac{N_{v}}{N_{h}}V S -(\mu_{h} + \gamma_{h})I,  \end{array} $$

3c $$\begin{array}{*{20}l} \frac{dR}{dt} = \gamma_{h} I - \mu_{h} R, \end{array} $$

3d $$\begin{array}{*{20}l} \frac{dU}{dt} = \frac{D}{N_{v}}-\mu_{v} U - \beta_{v}U I, \end{array} $$

3e $$\begin{array}{*{20}l} \frac{dV}{dt} = \beta_{v} UI - \mu_{v} V.  \end{array} $$

Ratio $\frac {N_{v}}{N_{h}}$ is the *per–capita vector density* and denoted by *n*. Since *S*=1−*I*−*R* and *V*=1−*U*, the system can be reduced to a three dimensional system:

4a $$\begin{array}{*{20}l} \frac{dI}{dt} = \beta_{h} n V(1-I-R) -(\mu_{h} + \gamma_{h})I, \end{array} $$

4b $$\begin{array}{*{20}l} \frac{dR}{dt} = \gamma_{h} I - \mu_{h} R, \end{array} $$

4c $$\begin{array}{*{20}l} \frac{dV}{dt} = \beta_{v} (1-V)I - \mu_{v} V. \end{array} $$

Recall the estimation of infected vector population is practically an infeasible task. Moreover, simulation of three dimensional system in Eq. (4) with certain assumptions provides infected vector population which cannot be validated. Therefore, three dimensional system further be simplified by considering quasi–equilibrium for infected vector population.

#### Quasi–equilibrium IR model

Since the time–scale of the vector population is much faster than the host [25], it is the vector dynamics which achieve the first equilibrium. Hence, we consider the infected vector population to be in its quasi–equilibrium [26]. Let $\beta = \frac {\beta _{v}}{\mu _{v}}$. Then the quasi–equilibrium values for vector population be given by *U*^∗^ and *V*^∗^.

5 $$ {U^{*}} = \frac{1}{\beta I + 1}, \hspace{8pt} {V^{*}} = \frac{\beta I}{\beta I + 1}.   $$

Substituting the quasi–equilibrium value *V*^∗^ for the infected vectors into Eq. (4a), it is possible to obtain the reduced two–dimensional quasi–equilibrium IR model as in Eq. (6).

6a $$\begin{array}{*{20}l} \frac{dI}{dt} = \beta_{h} n \frac{\beta I}{\beta I + 1} (1-I-R) - (\mu_{h} + \gamma_{h})I, \end{array} $$

6b $$\begin{array}{*{20}l} \frac{dR}{dt} =\gamma_{h} I - \mu_{h} R. \end{array} $$

Before using this two–dimensional dynamical quasi–equilibrium IR model, it is important to compare and verify there is no significant difference between two models in both qualitative and quantitative point of view under the same theoretical framework.

### Comparison of quasi–equilibrium IR model and classical SIR model

#### Comparison by simulation

**Fig. 3:**
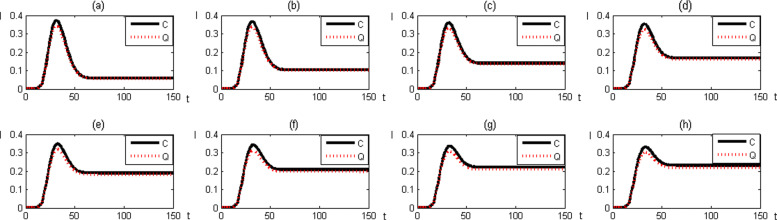
Comparison of the dynamical behaviour of the infected host population for **a** *β*_*h*_=0.05, **b** *β*_*h*_=0.1, **c** *β*_*h*_=0.15, **d** *β*_*h*_=0.2, **e** *β*_*h*_=0.25, **f** *β*_*h*_=0.3, **g** *β*_*h*_=0.35 and **h** *β*_*h*_=0.4. (I is Infected vector population, C is classical SIR model and Q is Quasi–equilibrium model)

In order to illustrate the dynamical behavior of both models described in system (4) and (6), numerical simulation was performed using *MATLAB ode45*. For this study we assume that parameters *μ*_*h*_, *μ*_*v*_ and *D* are constant for given time period. Sensitivity analysis with respect to parameters *β*_*h*_, *β*_*v*_ and *γ* are carried out numerically to determine the difference of the dynamical behavior of the infected host population in the quasi–equilibrium IR model with the reduced three dimensional SIR system. Figure 3 exhibits the comparison of the dynamical behavior of the infected host population in both models for different *β*_*h*_ values.

From Fig. 3, it can be observed that there is no significant difference between qualitative behaviour of infected host population. Moreover, we found that the difference between infected host populations is less than 5 per 1000 inhabitants. In other words, it illustrates that there is no significant difference between qualitative and quantitative behavior of infected host population under both models.

#### Comparison by stability

Now, we compare the equilibrium points and the stability statues of both models. First, we consider the reduced classical SIR model represented by system (4). The system admits two equilibrium points and for the simplicity we use ${R_{0}}=\frac {\beta _{h} \beta n}{\mu _{h} + \gamma _{h}} $, $P=\frac {\gamma _{h}}{\mu _{h}}$, $Q=\frac {\beta _{h}}{\mu _{h}}$ and $M =\frac {\mu _{h} +\gamma _{h}}{\mu _{h}} $. We assume that *R*_0_, *P* and *M* are constants. Here *R*_0_ is the basic reproduction number and *R*_0_ representing how many secondary infectious result from the introduction of one infected individual into a susceptible population. Trivial equilibrium of the system is given by (*I*^∗^,*R*^∗^,*V*^∗^)=(0,0,0) while non trivial equilibrium is given by *I*^∗^, *R*^∗^ and *V*^∗^.

7 $$ I^{*} = \frac{{R_{0}} - 1}{\beta(1 + Q n)}, \hspace{8pt} R^{*} = \frac{{P(R_{0}} - 1)}{\beta(1 +Q n)},\hspace{8pt} V^{*} = \frac{{R_{0}} - 1}{R_{0} +Q n}.   $$

Let us show that if the basic reproduction number *R*_0_ is less than unity, the disease free equilibrium is locally asymptotically stable. In order to analyze the stability of the equilibrium states, we look at the Jacobian matrix and its eigenvalues. The Jacobian at trivial equilibrium is given by,

8 $$ J_{(0,0,0)} =\left(\begin{array}{ccc} - (\mu_{h} + \gamma_{h}) & 0 & \beta_{h} n \\ \gamma_{h} & -\mu_{h} & 0\\ \beta_{v} & 0 & -\mu_{v} \end{array}\right).   $$

Then the characteristic polynomial of the *J*_(0,0,0)_ is,

9 $$ \begin{aligned} X_{1}(\lambda) = (\lambda+\mu_{h})\left(\lambda^{2} + \lambda(\mu_{h}+\mu_{v}+\gamma_{h})+\mu_{v}(\mu_{h}+\gamma_{h})(1-R_{0})\right). \end{aligned}  $$

From Eq. (9), it can be observed that one eigen value of *J*_(0,0,0)_ is negative. In order to investigate the local stability of the equilibrium state we consider the Routh–Hurwitz criterion for a second degree polynomial [27]. According to the Routh–Hurwitz criterion, polynomial *X*_1_ is locally asymptotically stable, if (*μ*_*h*_+*μ*_*v*_+*γ*_*h*_)>0 and *μ*_*v*_(*μ*_*h*_+*γ*_*h*_)(1−*R*_0_)>0. Clearly, *μ*_*v*_(*μ*_*h*_+*γ*_*h*_)(1−*R*_0_)>0 if *R*_0_<1. Hence, the trivial equilibrium (0,0,0) is locally asymptotically stable whenever *R*_0_<1.

Now consider the endemic equilibrium (*I*^∗^,*R*^∗^,*V*^∗^). The Jacobian matrix at (*I*^∗^,*R*^∗^,*V*^∗^) is given by,

10 $$ \begin{aligned} J_{\left(I^{*},R^{*},V^{*}\right)} =\left(\begin{array}{ccc} - \left(\beta_{h} v^{*} n +\mu_{h} + \gamma_{h}\right) \hspace{10pt} & -\beta_{h} v^{*} n \hspace{10pt} & \beta_{h} n(1-I^{*}-R^{*}) \\ \gamma_{h} & -\mu_{h} & 0\\ \beta_{v}(1-v^{*}) & 0 & -(\beta_{v} I^{*} +\mu_{v}) \end{array}\right).  \end{aligned}  $$

Then the characteristic polynomial corresponding to $\phantom {\dot {i}\!}J_{(I^{*},R^{*},V^{*})}$ is given by,

11 $$ X(\lambda) = \lambda^{3} + a_{1}\lambda^{2} + a_{2}\lambda+a_{3},   $$

where,

12a $$\begin{array}{*{20}l} a_{1} =& \frac{(\mu_{h}\!+\gamma_{h})(R_{0}+\beta+P)}{(\beta+P+1)} \!+ \mu_{h} \!+\mu_{v}\!+ \frac{\beta_{v}(R_{0}-1)}{(\beta+R_{0}+R_{0} P)}, \end{array} $$

12b $$\begin{array}{*{20}l} a_{2} =& \frac{(\mu_{h}+\gamma_{h})}{(\beta+P+1)}\left({\vphantom{\frac{\beta_{v} (R_{0} +\beta+P)(R_{0}-1)}{(\beta +R_{0} + R_{0} P)}}} \mu_{h}(R_{0} +\beta+P) +(R_{0}-1)\gamma_{h}\right. \\ &\left.+ \frac{\beta_{v} (R_{0} +\beta+P)(R_{0}-1)}{(\beta +R_{0} + R_{0} P)}\right) -\mu_{h}\beta_{v} P  \\ &+\frac{\mu_{h} \beta_{v} (R_{0}-1)}{(\beta + R_{0} +R_{0} P)}, \end{array} $$

12c $$\begin{array}{*{20}l} a_{3} =& \frac{(\mu_{h} +\gamma_{h})}{(\beta + P + 1)}\left(\frac{\mu_{h}\beta_{v}(R_{0}+\beta+P)(R_{0}-1)}{(\beta +R_{0} +R_{0} P}\right. \\ &\left.+(R_{0} +\beta + P)\mu_{v} {\vphantom{\frac{\beta_{v} (R_{0} +\beta+P)(R_{0}-1)}{(\beta +R_{0} + R_{0} P)}}}\right) \!+\frac{(\mu_{h} +\gamma_{h})}{(\beta + P + 1)}\left(\frac{\gamma_{h}\beta_{v}(R_{0}-1)^{2}}{(\beta + R_{0} +R_{0} P)} \right. \\ &\left.+\mu_{v}(R_{0}-1)-\mu_{h}\beta_{v}(\beta + P +1){\vphantom{\frac{\beta_{v} (R_{0} +\beta+P)(R_{0}-1)}{(\beta +R_{0} + R_{0} P)}}}\right).  \end{array} $$

According to Routh–Hurwitz criterion for third order polynomial, the equilibrium point (*I*^∗^,*R*^∗^,*V*^∗^) is locally asymptotically stable if the polynomial satisfies the condition given in (13).

13 $$ a_{1} >0, \hspace{8pt} a_{2} >0 \hspace{8pt} \text{and} \hspace{8pt} a_{1} a_{2} >a_{3}.  $$

From Eqs. (12a) and (12b), it can be observed that *a*_1_>0 and *a*_2_>0 whenever *R*_0_>1. Furthermore, *a*_1_*a*_2_>*a*_3_ if *R*_0_>1. Therefore, the equilibrium (*I*^∗^,*R*^∗^,*V*^∗^) locally asymptotically stable if *R*_0_>1.

Now let us consider the reduced quasi–equilibrium IR model in (6). The system admits two equilibrium points. Virus free state of the system is given by $\left (\hat {I},\hat {R}\right) =(0,0)$ while non trivial equilibrium is given by $\left (\hat {I},\hat {R}\right)$ where,

14 $$ \hat{I} = \frac{{R_{0}} - 1}{\beta +R_{0} M}, \hspace{8pt} \hat{R} = \frac{{P(R_{0}} - 1)}{\beta +R_{0} M}.   $$

Let us show if the basic reproduction number (*R*_0_) less than unity, the disease–free equilibrium is locally asymptotically stable and if *R*_0_>1, the endemic equilibrium is locally asymptotically stable. The Jacobian at trivial equilibrium is given by,

15 $$ J_{(0,0)} = \left(\begin{array}{ccc} \beta_{h} \beta n - (\mu_{h} + \gamma_{h}) & 0 \\ \gamma_{h} & -\mu_{h} \end{array}\right).   $$

Notice that eigenvalues of *J*_(0,0)_ are (*R*_0_−1)(*μ*_*h*_+*γ*_*h*_) and −*μ*_*h*_. If *R*_0_<1, then both eigenvalues are negative. So the trivial equilibrium state is stable for *R*_0_<1. If *R*_0_>1, (*R*_0_−1)(*μ*_*h*_+*γ*_*h*_) is positive. Then *J*_(0,0)_ has one positive and one negative eigenvalue. So the system is unstable for *R*_0_>1.

Now consider the Jacobian at non–trivial $\left (\hat {I},\hat {R}\right)$ given by,

16 $$ J_{\left(\hat{I},\hat{R}\right)} = \left(\begin{array}{ccc} - \frac{\beta_{h} n ({R_{0}}-1)(R_{0} +\beta)}{R_{0}({R_{0}} + Mn)} & \frac{-\beta_{h} n ({R_{0}}-1)}{(R_{0} +Mn)} \\ \gamma_{h} & -\mu_{h} \end{array}\right).   $$

Then the characteristic polynomial of $J_{\left (\hat {I},\hat {R}\right)}$ is,

17 $$ Y(\lambda) = \lambda^{2} + b_{1}\lambda + b_{2},   $$

where, $b_{1} = 1 + \frac {{R_{0}}M({R_{0}}-1)}{\beta ({R_{0}} +Pn) + \frac {({R_{0}}-1)M}{(Pn+1)}}$ and $b_{2} = \frac {M({R_{0}}-1)}{({R_{0}} +Pn)}$. Due to the Routh–Hurwitz criterion for a second degree polynomial, the polynomial *Y* is locally asymptotically stable if *b*_1_>0 and *b*_2_>0. Therefore, $\left (\hat {I},\hat {R}\right)$ is locally asymptotically stable if and only if *R*_0_>1.

It is important to note that both models have the same basic reproduction number (*R*_0_). However, the dimension of the quasi–equilibrium IR model is less than the reduced SIR compartmental model. Thus, computational cost can be reduced by using quasi–equilibrium IR model. Moreover, parameter estimation can be done efficiently using the derived simple model. Hence, for this study we use quasi–equilibrium IR model instead of reduced SIR compartmental model.

## Development of a data-driven mathematical model

As mentioned in the introduction, *per–capita vector density* (*n*) depends upon climate factors. Since climate factors change with time, *n* is a time dependent parameter. Hence, we have to analyze reported dengue data and meteorological data in CMC area to define a function for *n* to capture the seasonal effect of the disease.

### Data analysis

In literature many studies mentioned that temperature and precipitation cause prominent effects on periodic pattern of dengue dynamics [3, 11, 12, 28, 29]. Hence, we use rainfall data, maximum temperature and minimum temperature to capture the periodic pattern of dengue dynamics. Notice that dengue incidences and meteorological data have been recorded in *time domain*, and, for the pattern identification, we need to extract *frequency domain* features. Hence, fast Fourier transformations has been used to convert time domain data into frequency domain.

To determine lag times, the cross correlation between dengue incidences and climate data are computed. For that purpose we use Pearson cross–correlation formula.

For our study we have considered weekly reported dengue incidents gained from Epidemiology unit, Department of Health, Sri Lanka, weekly rainfall, maximum temperature and minimum temperature data from Meteorological Department, Sri Lanka from 2009 to 2015 for CMC area.

### Fourier analysis

**Fig. 4:**
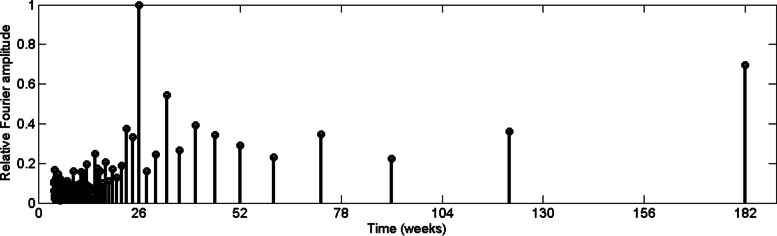
Relative Fourier spectrum value of reported dengue incidence in CMC area

Fourier analysis has been used in many fields for pattern identification [30]. Fast Fourier transform extracts the frequency information of a time series. The discrete Fast Fourier transform for the time series {*x*_*n*_} represented by Eq. (18) is obtained by decomposing a sequence of values into components of different frequencies. In our context, frequencies are reciprocal of number of weeks. In this section we discuss the pattern of dengue data, rainfall data, maximum temperature and minimum temperature data reported on CMC area from 2009 to 2015 using Fourier spectrum *A*(*k*) at *k*th frequency,

18 $$ A(k)= \sum\limits_{n=0}^{N-1} \exp{\left(-i\frac{2\pi}{N}kn\right)}x_{n},  $$

where, *N* is number of data points in time series {*x*_*n*_}. For the purpose of comparison we compute the relative Fourier transform by dividing all the spectrum by highest Fourier amplitude in the spectrum. In order to examine the periodic pattern of the reported dengue incidences, relative Fourier spectrum for CMC weekly dengue data from 2009 to 2015 has been computed, which is depicted in Fig. 4.

**Fig. 5:**
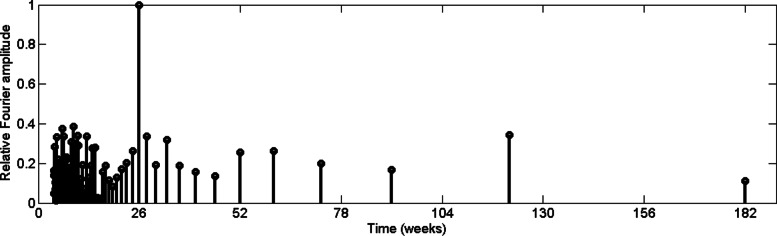
Relative Fourier spectrum value of rainfall data in CMC area

According to Fig. 4, reported data from urban Colombo exhibit a 26–week periodic pattern. In other words, it illustrates that dengue cases increase in every six and half months. In addition, a Fourier amplitude related to two and half year periodic pattern can be observed with 0.7 relative amplitude. It should be noted that dengue outbreak has a 2–3 year cycle [3] and the serotype shift may have contributed the cycling of outbreaks. Then we computed the relative Fourier spectrum for rainfall data for the same time period, which is depicted in Fig. 5.

From Fig. 5, it can be noticed that rainfall data from urban Colombo show a 26–week periodic pattern. As we mentioned in the introduction, Colombo is influenced by two monsoon seasons and there are two friendly climate seasons for mosquitoes within a year. Thus, the 26–week period of rainfall underscores its correlation with dengue incidences.

**Fig. 6:**
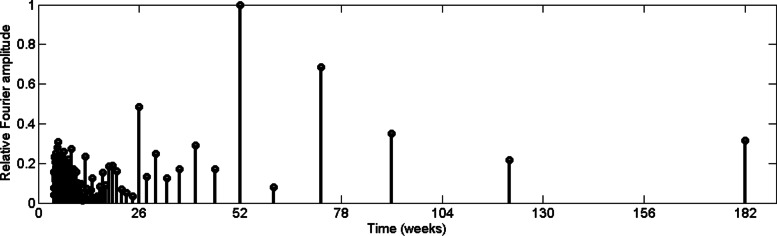
Relative Fourier spectrum value of maximum temperature in CMC area

**Fig. 7:**
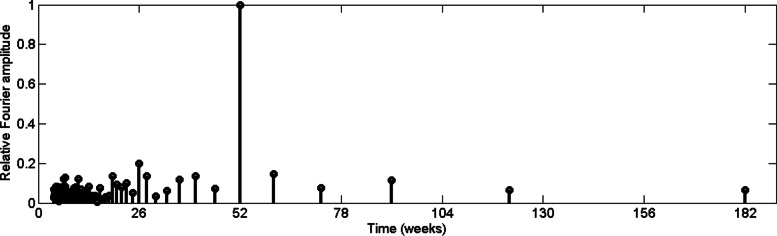
Relative Fourier spectrum value of minimum temperature in CMC area

Similarly, we examined periodic pattern of maximum temperature data and minimum temperature from 2009 to 2015, which are depicted in Figs. 6 and 7.

According to Figs. 6 and 7 it can be observed that, temperature data exhibit a 52–week periodic pattern. In other words, results indicate that maximum temperature and minimum temperature in urban Colombo have annual pattern. In addition, it can be observed from Fig. 6, a Fourier amplitude related to 26–week with 0.5 relative amplitude. It should be noted that temperature differs depends on rainfall [31] and the rainfall pattern may have contributed the maximum temperature pattern.

After the pattern analysis, our next task is to determine the time lags between dengue incidence and climate factors.

### Correlation analysis

Notice that *Aedes* mosquitoes proceed life cycle from eggs to adult through larvae and pupae and the life cycle takes approximately 1–2 weeks or longer depending on temperature, availability of water and nutrients [28]. Another interesting fact about dengue transmission is that the dengue mosquito eggs can withstand without desiccating for several months until it receives favorable conditions [32]. Also for infected humans, the incubation period ranges from 3 to 14 days. Those factors motivated and thrived us to measure the time delay of dengue infection with rainfall.

Moreover, *Aedes* mosquitoes emerge from eggs to adults in a shorter period at higher temperature and the mortality rates of adult mosquitoes increase with increasing temperature above 30^∘^*C* [29]. Furthermore, infected human experience a shorter incubation period for dengue viruses with high temperature [28]. Those factors motivated us to calculate the time delay of reported dengue incidences with maximum temperature.

In order to calculate the time delay, the Pearson correlation formula has been used. The Pearson correlation formula for two time series *Z*_1_ and *Z*_2_ can be represented by (19).

19 $$ \rho(Z_{1},Z_{2}) =\frac{cov(Z_{1},Z_{2})}{\sigma(Z_{1})\sigma(Z_{2})}.  $$

Here, *cov*(*Z*_1_,*Z*_2_) denotes the covariance of the variables *Z*_1_ and *Z*_2_. Standard deviation of *Z*_1_ and *Z*_2_ denoted by *σ*(*Z*_1_) and *σ*(*Z*_2_).

**Fig. 8:**
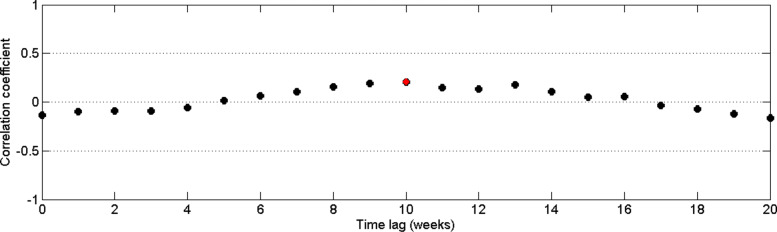
Pearson’s cross–correlation of dengue data with rainfall data in CMC area

The correlation measure between dengue infection and rainfall was plotted with time lags from 0 to 20 weeks. Here we assume that the disease occurs within or after rainy seasons. Figure 8 represents the correlation measures between weekly rainfall data and weekly dengue data for the period of 2009 to 2015 in CMC area with time delay.

By Fig. 8, it can be observed that the highest correlation occurs with a 10–week delay. Since the highest correlation value is less than 0.5, correlation measures against time lags from 0 to 20 weeks were plotted for each year from 2009 to 2015 to calculate the annual time lag.

**Fig. 9:**
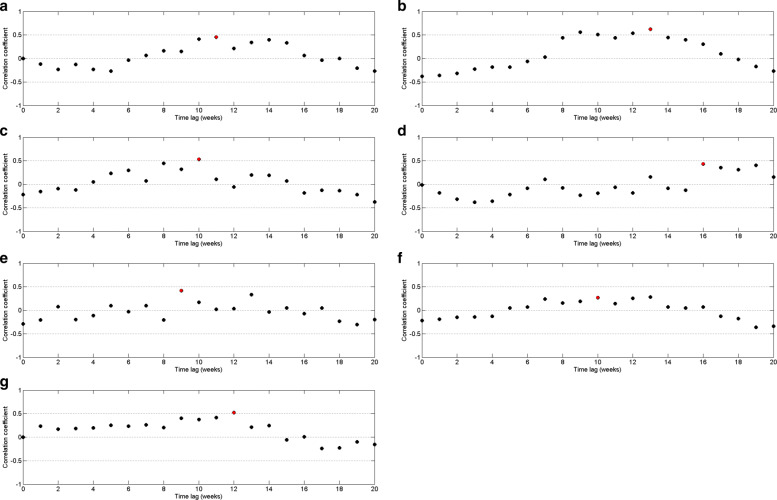
Pearson’s cross–correlation of dengue data with rainfall data in CMC area for **a** 2009 **b** 2010 **c** 2011 **d** 2012 **e** 2013 **f** 2014 and **g** 2015

According to correlation values between dengue and precipitation, it can be observed by Fig. 9 that precipitation has a delayed effect of 8 to 16 weeks on dengue. This pattern is common for each year except year 2012, which implies that most of the dengue cases reported with 8 to 16 weeks delay with rainfall. In year 2012, the correlation between rainfall and dengue incidences shows a delayed effect of 16 to 19 weeks.

### Rainfall pattern in CMC area and cutoff values for rainfall data

As mentioned in the introduction rainfall pattern in Sri Lanka is governed by its tropical location and the monsoon seasons and consequently has a strong seasonal variation in the rainfall pattern. CMC area belongs to the wet zone with 2500*mm* average annual rainfall and 75% of average rainfall occurs during the two monsoon seasons [33]. According to the statistics for Colombo from 2009–2015, the number of annual rainy days ranges from 133 to 208 days. That is, precipitation occurs on more than 36% of the total days in a year. Hence, rainfall pattern in Colombo provides an ideal environment for vectors [11].

Although monsoon seasons provide ample breeding habitats for *Aedes* mosquitoes, heavy rainfall can potentially flush away larvae or pupae or the immature stage of mosquitoes [34, 35]. In addition, heavy rainfall can increase the mortality rate of adult mosquitoes[11]. According to [33], rainfall pattern in urban Colombo has positive association with extreme rainfall events and identified as one of the flood risk area in Sri Lanka. Hence, decrease the spread of dengue transmission in the period with extreme rainfall. Moreover, El Niño Southern Oscillation (ENSO) influences the seasonal variability of rainfall, specially in the tropical zone of the world [36]. Sri Lanka experience an excess of seasonal rainfall during El Nino years [36] and consequently, influence the vector populations. Therefore, finding the weekly rainfall value, which can make favorable environment for dengue is important for decision makers to predict the number of dengue incidences in upcoming monsoon seasons. To calculate the weekly rainfall value, which gives highest correlation value with dengue data, we used minimum and maximum cutoff values on rainfall data.

**Fig. 10:**
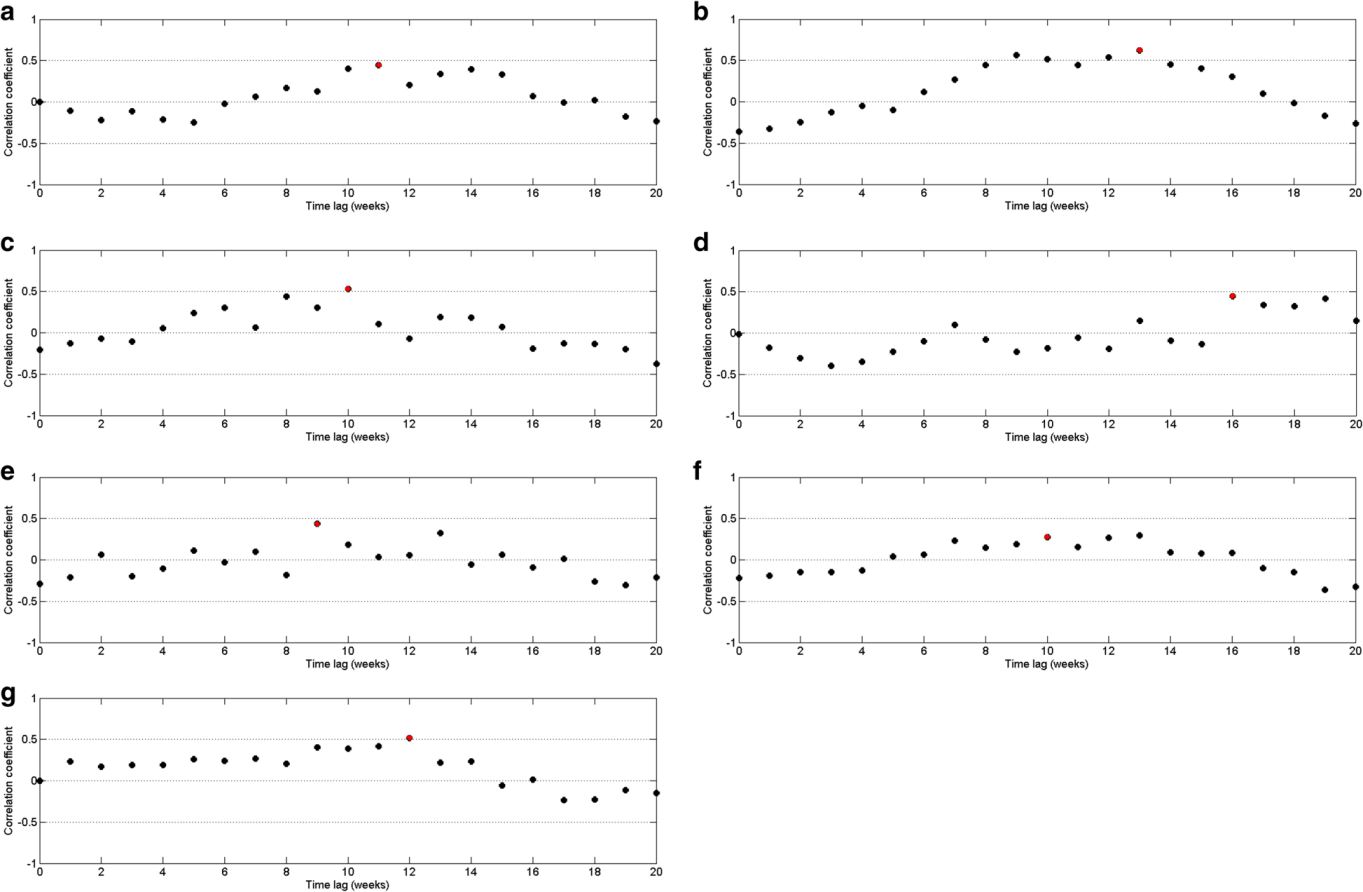
Pearson’s cross–correlation of dengue data with rainfall data within cutoff values in CMC area for **a** 2009 **b** 2010 **c** 2011 **d** 2012 **e** 2013 **f** 2014 and **g** 2015

For each year of period from 2009 to 2015, we evaluated highest correlation value and time lag by increasing reported minimum rainfall value and decreasing maximum rainfall value. Then we found the best match minimum and maximum cutoff values are 14*mm* and 454*mm* weekly rainfall with 10 weeks delay. Moreover, we observed that more than 65% of data values fall within the interval of 44*mm* to 454*mm*. However, the cutoff values for total time period gives 0.24 for the highest correlation value. Therefore, correlation between dengue and rainfall data has been calculated for each year separately using the rainfall values within minimum and maximum cutoff values. Figure 10 represents the correlation of dengue data with rainfall data within cutoff values. It demonstrates that weekly rainfall value between 14*mm* to 454*mm* influences the risks of dengue cases at lag times 8–weeks up to 12–weeks with higher relative risks. This pattern is common for every year between 2009 to 2015 except 2012.

**Fig. 11:**
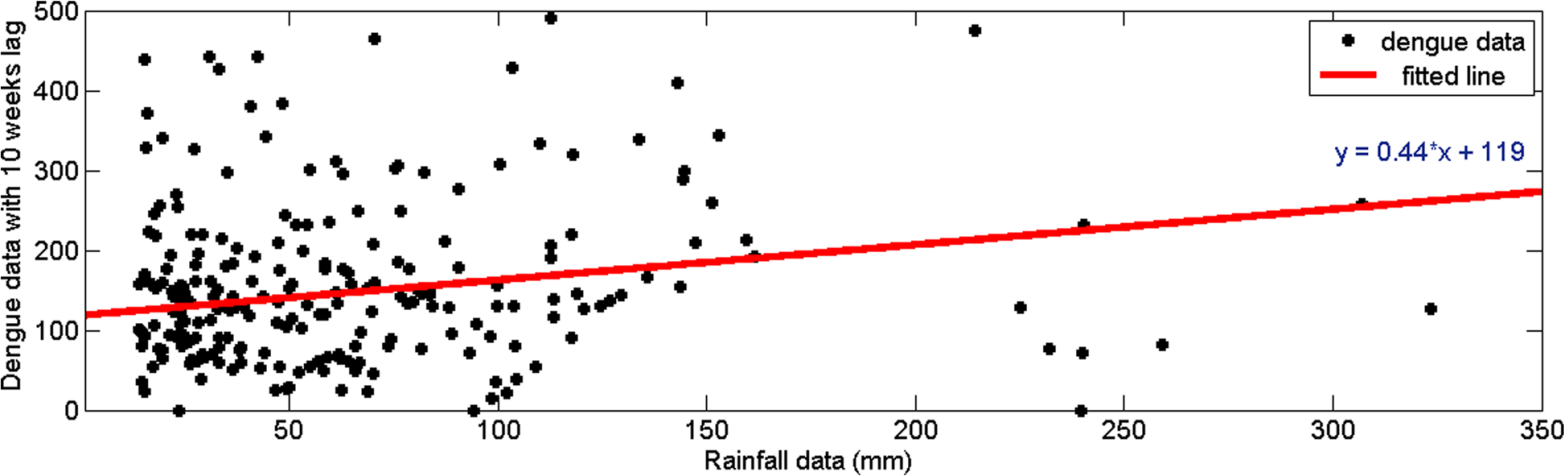
Scatter plot of dengue data and rainfall data with 10–weeks time delay in CMC area

In addition, Fig. 11 shows the distribution of dengue incidence and rainfall data with 10–weeks lag time in Colombo Municipal Council area.

**Fig. 12:**
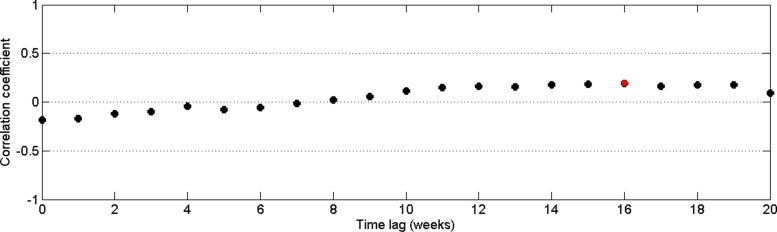
Pearson’s cross–correlation of dengue data with maximum temperature data in CMC area

**Fig. 13:**
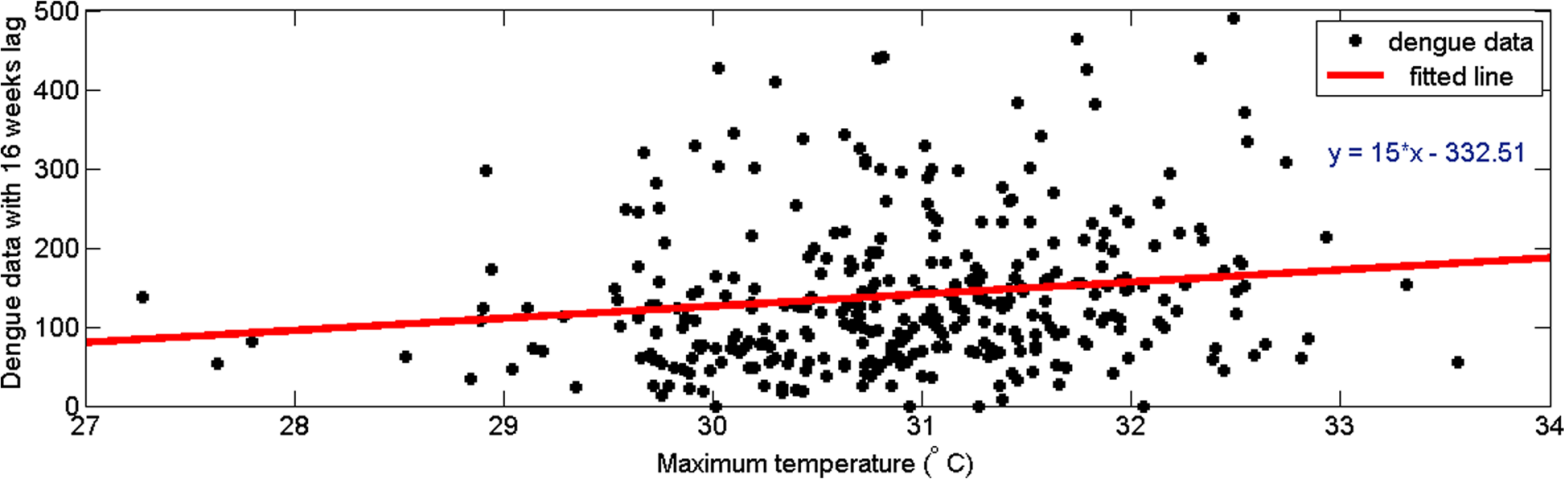
Scatter plot of maximum temperature data with dengue data with 16–weeks time delay in CMC area

Now consider the time delay of reported dengue incidences with maximum temperature. It can be observed from Fig. 12 that the highest correlation occurs with a 16–week delay. Figure 13 represents the distribution of dengue data and maximum temperature data with 16–week delay. Since the highest correlation value is less than 0.2, correlation measures against time lags from 0 to 20 weeks were plotted for each year from 2009 to 2015 to calculate the annual time lag.

**Fig. 14:**
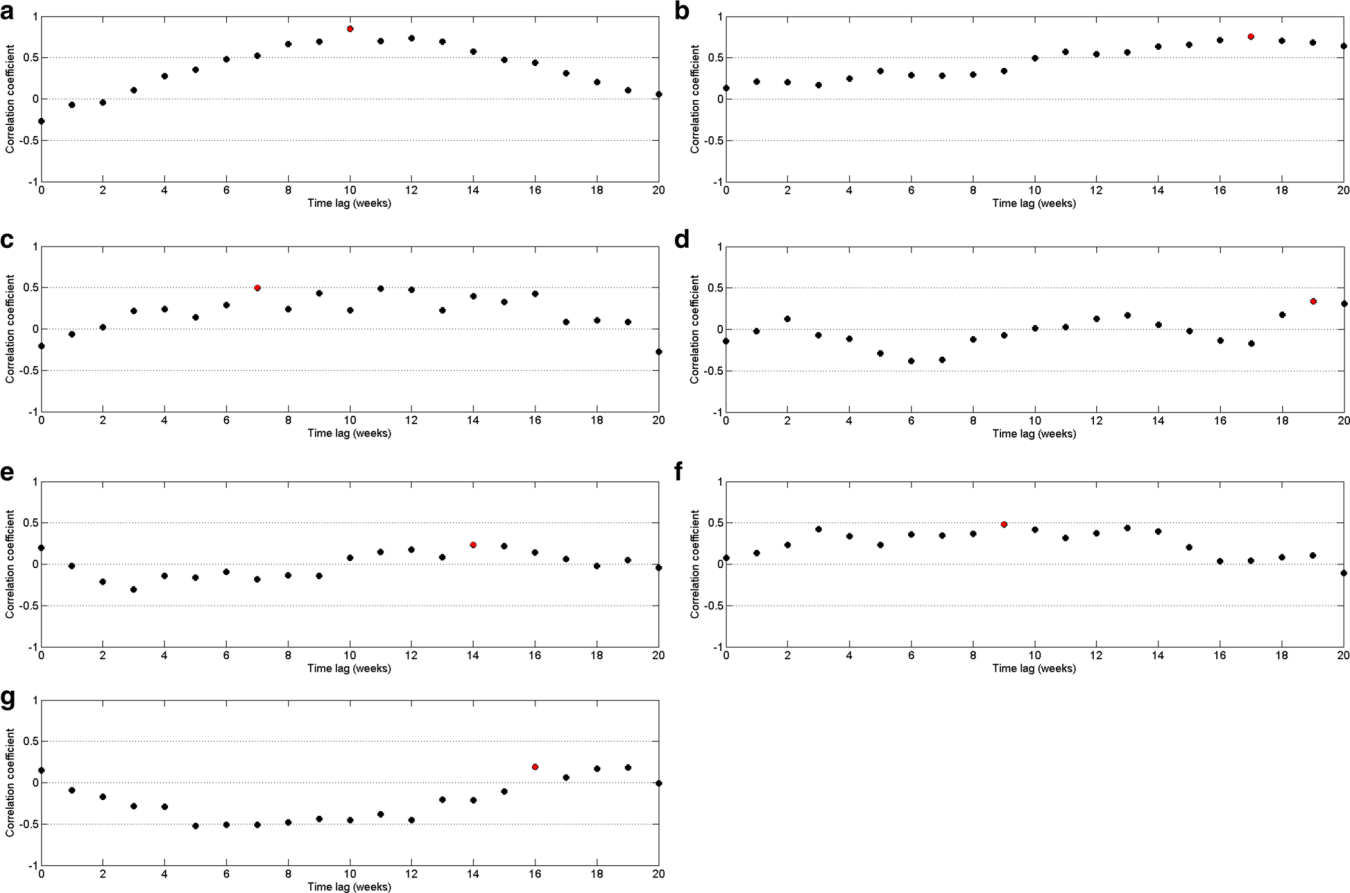
Pearson’s cross–correlation of dengue data with maximum temperature data in CMC area for **a** 2009 **b** 2010 **c** 2011 **d** 2012 **e** 2013 **f** 2014 and **g** 2015

From Fig. 14, it can be observed that there is no common correlation pattern or lag period. Only for year 2009 and 2010 highest correlation value between maximum temperature and dengue is grater than 0.5. Since ENSO influenced on local temperature and precipitation worldwide, we consider the ENSO years and intensities [37] to clarify the effect on correlation between dengue and maximum temperature. According to ENSO years and intensities [37] year 2009, 2010 had moderate *El–nino* effects and during the event temperature was increased. ’This event might have caused mosquitoes to emerge over a shorter period of time, and the virus to have a shorter (extrinsic) incubation period. Further, it can be seen a negative correlation till 16th weeks lag time in year 2015. Year 2015 had strong *El–nino* effect and it might have caused to increase the mortality rates of adult mosquitoes.

**Fig. 15:**
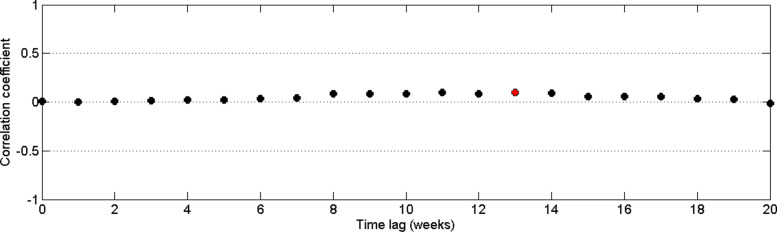
Pearson’s cross–correlation of dengue data with minimum temperature data in CMC area

**Fig. 16:**
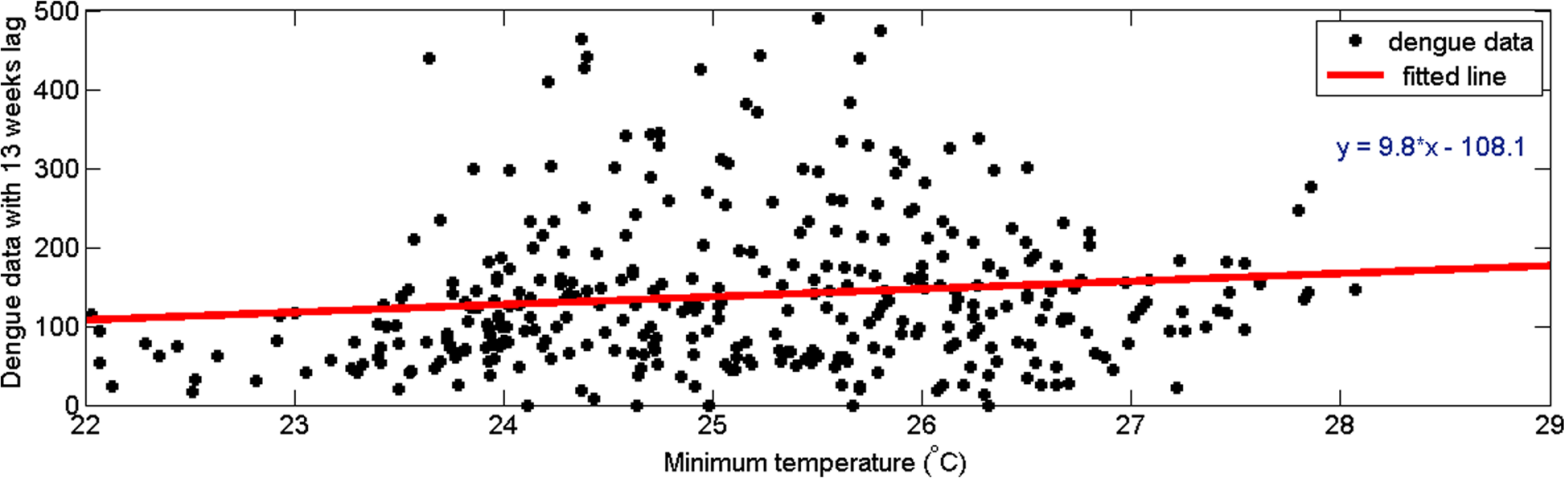
Scatter plot of minimum temperature data with dengue data with 13–weeks time delay in CMC area

Similarly, we analyzed time delay of dengue incidences with minimum temperature. From Fig. 15, it can be observed that the highest correlation occurs with a 13–week delay. In addition, from Fig. 16, it can be observed the distribution of dengue data and minimum temperature data with 13–weeks delay. Since the highest correlation value is less than 0.1, correlation measures against time lags from 0 to 20 weeks were plotted for each year from 2009 to 2015 to calculate the annual time lag.

**Fig. 17:**
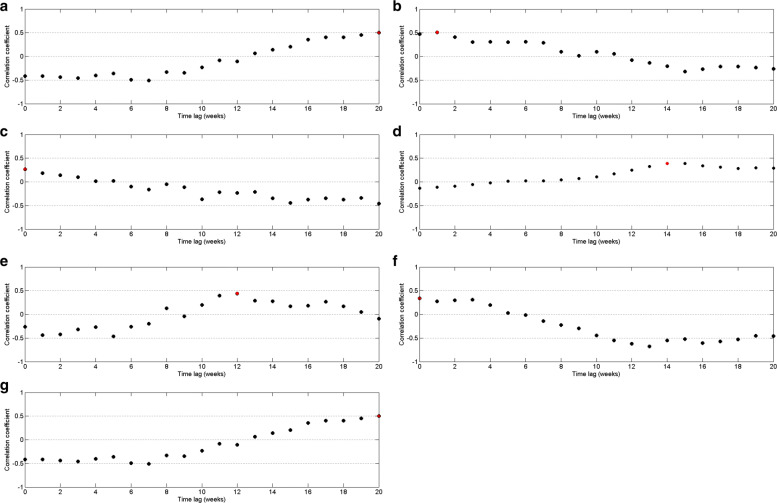
Correlation of dengue data with minimum temperature data in CMC area for **a** 2009 **b** 2010 **c** 2011 **d** 2012 **e** 2013 **f** 2014 and **g** 2015

Figure 17 represents the correlation values between minimum temperature and dengue incidences against time lag. In years 2009 and 2010, correlation between dengue incidences and minimum temperature is positive until 10 weeks time lag. In year 2015, correlation between minimum temperature and dengue reported cases begin to increase with 10 weeks time delay.

### Parameters estimation model

From data analysis results, it can be observed that seasonality pattern and time delay of the disease depend upon climate factors. Hence, rainfall data, maximum temperature data and minimum temperature data with time delay can be used to capture the periodic pattern and magnitude of *per–capita vector density*. Moreover, the infected vector density depends on infected human population. Hence the function for *n* can be defined as.

20 $$ \begin{aligned} n(t) =a_{1} d(t-l_{1}) + a_{2} pr(t-l_{2}) +a_{3} maxT(t-l_{3}) + a_{4} minT(t-l_{4}),  \end{aligned}  $$

where *d*, *pr*, *maxT* and *minT* denote reported dengue incidences, rainfall data, maximum temperature and minimum temperature data at time *t*. In our study *t* corresponds to the time in weeks. Therefore, we include time lag *l*_1_, *l*_2_, *l*_3_ and *l*_4_ to reported dengue incidences, rainfall, maximum temperature and minimum temperature variables respectively. *a*_1_, *a*_2_, *a*_3_ and *a*_4_ are coefficients constants. Based on the pattern and correlation analysis, we modified our formula in Eq. (20) accordingly. We used rainfall data with 10–weeks time delay, maximum temperature data with 16–weeks time delay, minimum temperature data with 13–weeks time delay and reported dengue data with 4–weeks time delay to define a function for *per–capita vector density*. Moreover, we used relative Fourier spectrum results to determine constant coefficients *a*_1_,*a*_2_,*a*_3_ and *a*_4_. Equation 21 represents the modified formulation for *per–capita vector density*.

21 $$ \begin{aligned} n(t)\! =a_{1} d(t-4) \!+ a_{2} pr(t-10) \!+a_{3} maxT(t-16) \!+ a_{4} minT(t-13).  \end{aligned}  $$

### Data driven model

Now the data driven quasi–equilibrium IR model can be read as,

22a $$\begin{array}{*{20}l} \frac{dI}{dt} =& \beta_{h} n(t) \frac{\beta I}{\beta I + 1} (1-I-R) - (\mu_{h} + \gamma_{h})I, \end{array} $$

22b $$\begin{array}{*{20}l} \frac{dR}{dt} =&\gamma_{h} I - \mu_{h} R, \end{array} $$

22c $$\begin{array}{*{20}l} \text{with} \\ n(t) =&a_{1} d(t-4) + a_{2} r(t-10) +a_{3} maxT(t-16) \\ &+ a_{4} minT(t-13). \end{array} $$

Notice that the data driven model in Eq. (22) is two dimensional model with real time data dependent parameter *n*(*t*). The model can be used to identify the dynamics of dengue in 4 weeks advance using real time data.

### Model validation

Real time data driven models should be able to predict the dynamics of the disease with sufficient time duration to take control measurements. For an example, WHO recommended to start adulticidal activities, specially space spraying treatments to control the disease transmission when first few dengue cases are detected or an outbreak is forecasted [2, 38]. The adulticidal treatments are valid for approximately two weeks period [38] and if adulticidal treatments are started early in an epidemic with sufficiently large scale then the intensity of transmission is reduced [2]. Hence, according to control point of view, predictive strength in an interval of two weeks is sufficient. Therefore, to measure the accuracy of the data driven quasi–equilibrium IR model in the interval framework, we consider the interval of [*t*−1,*t*+1] and define an error function *e*_*r*_(*t*) as,

23 $$ e_{r}(t) \!= min \left\{|I(t)-d\left(t'\right)|\frac{100,000}{N_{h}} | t' \in \{t-1,t,t+1\} \right\}.  $$

Here, *I*(*t*) and *d*(*t*) denote predicted data and actual data at time *t* and total population respectively. Notice that, *e*_*r*_(*t*) denotes relative error at time *t* and based on the relative error values, we define level of accuracy of the model *Ac*(*t*) as,

24 $$ Ac(t) =\left\{\begin{array}{ll} \text{highly significant} & \quad \text{if}\ e_{r}(t) <5, \\ \text{significant} & \quad \text{if}\ 5 \leq e_{r}(t) <10, \\ \text{average} & \quad \text{if}\ 10 \leq e_{r}(t) <15, \\ \text{poor} & \quad \text{if}\ 15 \leq e_{r}(t). \end{array}\right.  $$

## Numerical results

**Fig. 18:**
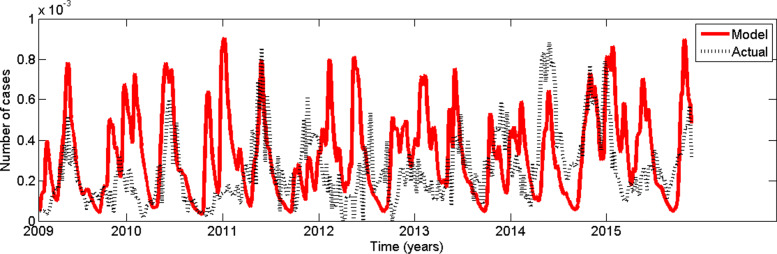
Comparison of actual dengue incidence with predicted number of cases from February, 2009 to December, 2015

In order to simulate the model (22) numerically, we use differential equation solver *ode45* in *MATLAB*. Table 1 represents the parameter values use in the model simulation. The function *ode45* implements a Runge–Kutta method. Figure 18 represents comparison between simulation results and reported dengue data. Analyzing the results, we can observe that simulated results captures the seasonality of the disease.

**Table 1:** Descriptions and values of all parameters used in the model simulation

Parameter	Definition	Value	Reference
*β*_*h*_	transmission rate for vector to host	0.75	[39]
*β*_*v*_	transmission rate for host to vector	0.375	[39]
*μ*_*h*_	reproduction and mortality rate of host	$\frac {1}{75 \hspace {4pt} \text {years}}$	[40]
*μ*_*v*_	reproduction and mortality rate of vector	$\frac {1}{4 \hspace {4pt} \text {days}}$	[39]
*γ*_*h*_	recovery rate of host	$\frac {1}{14 \hspace {4pt} \text {days}}$	[39]

Recall that the model depends on weekly reported dengue incidences, rainfall data, maximum temperature and minimum temperature with 4, 10, 16 and 13 weeks time lags respectively. Hence the model can be used to forecast of dengue incidences upto 4 week ahead using real time data.

**Fig. 19:**
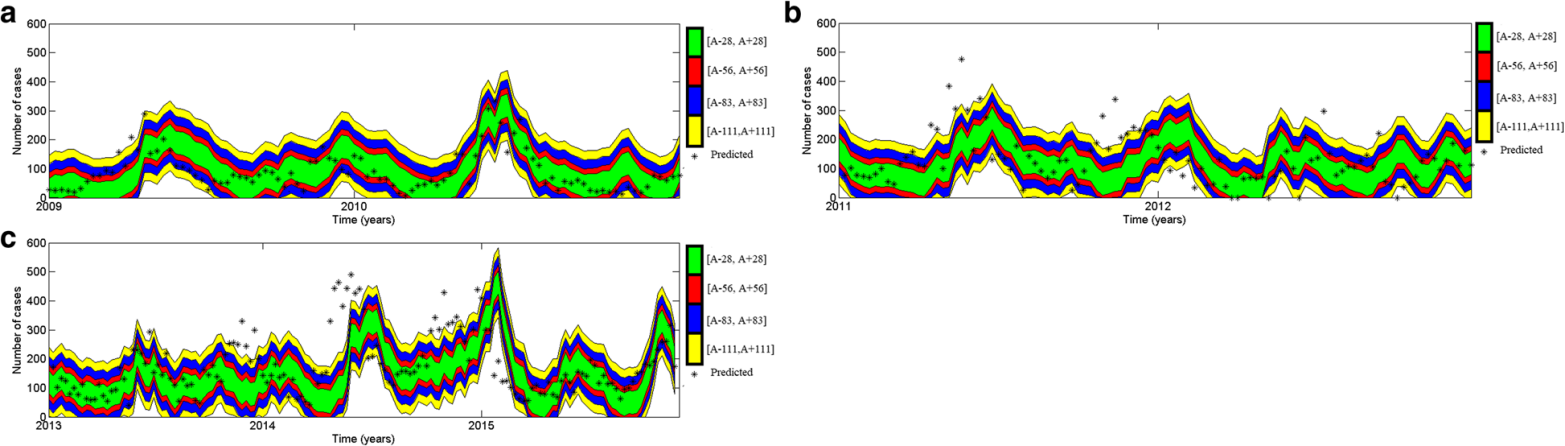
Accuracy level boundaries of the model **a** for year 2009 and 2010 **b** for year 2011 and 2012 and **c** for year 2013 to 2015 and predicted data from February,2009 to December, 2015. Notice that, 28, 56, 83 and 111 equivalent to 5, 10, 15 and 20 per 100,000 inhabitants (*A* is reported dengue data)

Then we investigate the level of accuracy of our model using error function in Eq. (23) and accuracy levels in Eq. (24). Figure 19 represents the accuracy level of the model from year 2009 to 2015.

**Fig. 20:**
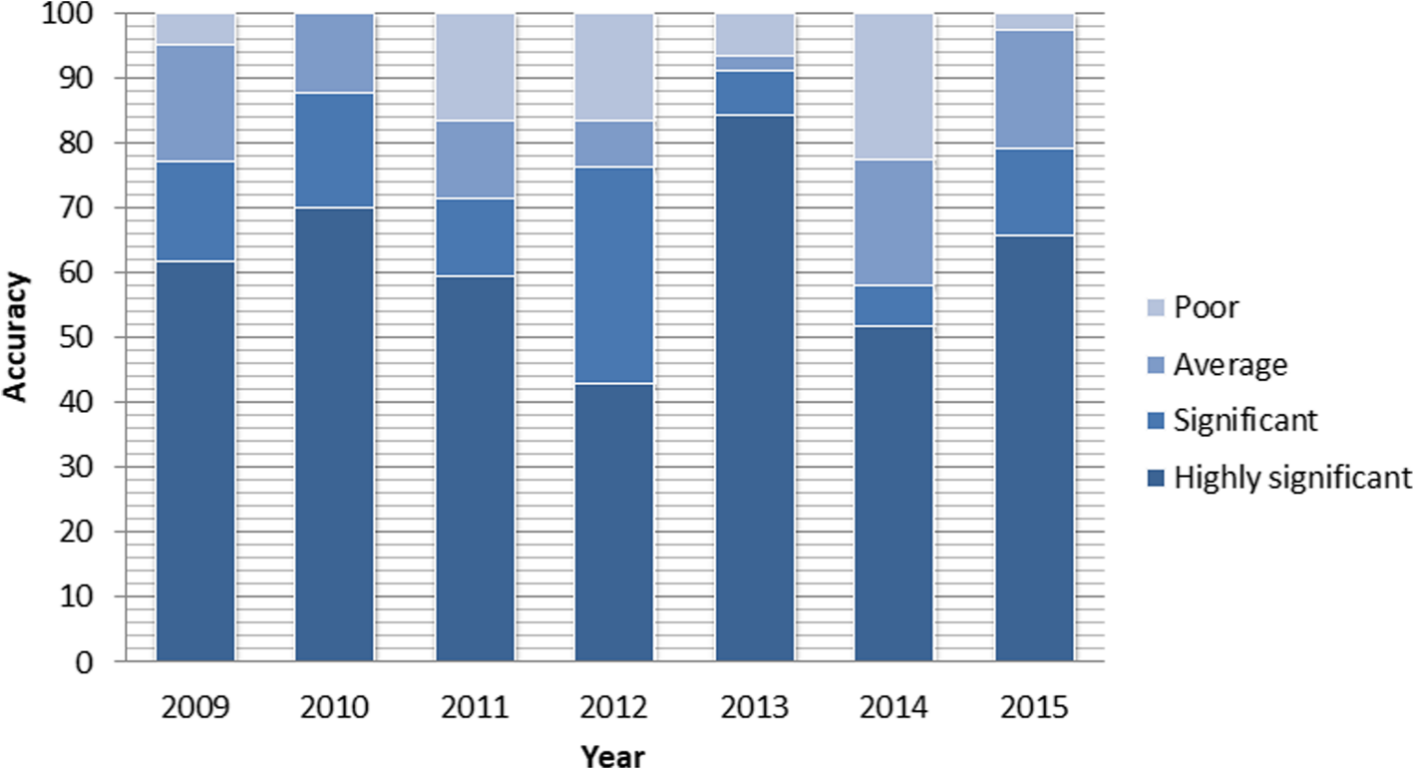
Annual results for the accuracy level of the model from year 2009 to 2015

From Fig. 19, it can be observed that most of actual dengue incidences data points lie within highly significant or significant accuracy level boundaries. Furthermore, for each year of period from 2009 to 2015, we analyzed the accuracy level of the model. The results shown graphically in Fig. 20 and numerically in Table 2.

**Table 2:** Annual results for the accuracy level of the model

Year	Highly Significant (*%*)	Significant (*%*)	Average (*%*)
2009	62	77	95
2010	70	88	100
2011	60	71	83
2012	44	77	84
2013	85	91	94
2014	52	58	77
2015	67	79	97

It is observed from the analysis that the level of accuracy of the model is 77%, 88%, 71%, 77%, 91%, 58% and 79% significant from year 2009 to 2015 respectively.

## Discussion

Developed data driven compartmental model predicts the dengue outbreak. The behaviour of dengue transmission significantly depends on many external variables including climatic factors. In this study we started with the classical compartmental model and reduced the model in to a quasi–equilibrium IR model. Then we compared the dynamical behaviour and the stability of the developed model with the classical model and identified that there is no significant difference between both models. Several previous works focused to developed theoretical models to determine the dynamic behaviour of dengue transmission [16, 41, 42]. However, the theoretical model may not provide the realistic results when the nature of the disease is highly diverse. Therefore, data driven compartmental models offer a promising direction, especially with the variables with potential impact on dengue transmission.

To capture the realistic nature of the disease, we developed the data driven quasi–equilibrium IR model. In the literature, a number of studies have been conducted to determine the effects of climate factors on the transmission of dengue in the context of mathematical and statistical models [12, 43–45]. Most of them have been concluded that the rainfall, temperature and humidity are the most important variables with potential impact on dengue transmission. However, the effect of climate factors on dengue transmission depends on regional location. Therefore, we can not use the results in these studies directly in our model and it is needed to develop a regional specified data driven model to predict dengue outbreak.

**Fig. 21:**
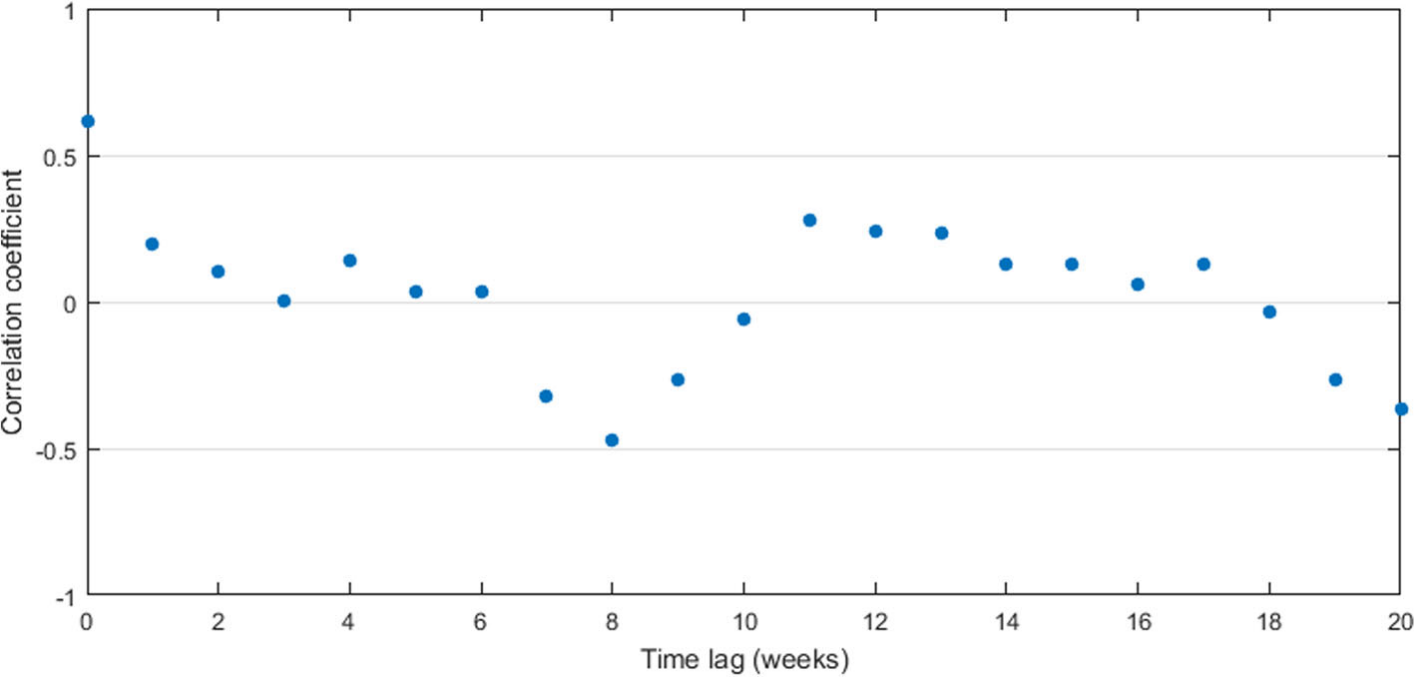
Pearson’s cross–correlation of relative humidity data with rainfall data in CMC area

**Fig. 22:**
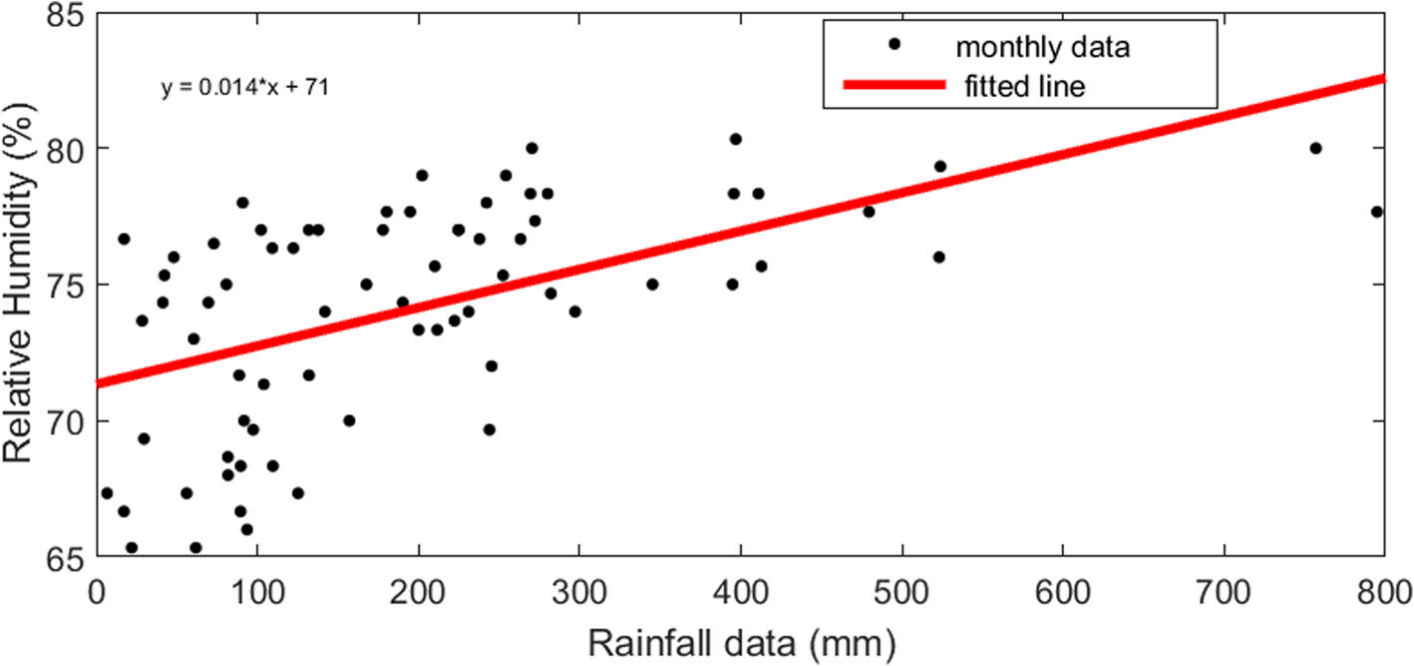
Scatter plot of relative humidity data with rainfall data with no time delay in CMC area

In a tropical country like Sri Lanka has the ideal temperature and relative humidity throughout the year [46] which create favorable environment to propagate the dengue transmission. As stated previously, the seasonal fluctuations of the disease in Sri Lanka have been governed by the precipitation data. From Fig. 21 it can be observed that the correlation between precipitation and relative humidity is higher than 0.5 and there is no delay effect between rainfall and relative humidity. That is humidity and rainfall move together and hence the effect of humidity on dengue transmission is captured by the rainfall data. The distribution of relative humidity data and rainfall data is illustrated in Fig. 22. In addition, the availability of temperature data is higher than the availability of relative humidity data. Therefore, we selected the rainfall data and temperature data as the climate factors for our model.

Based on reported dengue incidences, rainfall data and temperature data, we analyzed periodic and seasonal pattern of dengue infection for urban Colombo, Sri Lanka. Fourier spectrum over empirical data and precipitation data of CMC area have been indicated 26 weeks periodic pattern which differ from the situation in the city of Semarang (dengue data in the city of Semarang (Indonesia) shows annual periodic pattern [12]). The results demonstrated the seasonal pattern of dengue transmission depends on regional locations.

Moreover, we analyzed the correlation of the climate data using Pearson correlation coefficient. The result illustrated that the rainfall data, maximum temperature and minimum temperature data in CMC area have maximum correlation with dengue data with 10, 16 and 13 weeks lag respectively. Comparing the results with other studies [44, 47, 48], it can be observed that the predominant effect of the climate data also depends on the regional location. For an example, highest correlation between minimum temperature data and dengue incidence in Guadeloupe (French West Indies) occurs with a 5–week delay which differ from the situation in CMC area in Sri Lanka.

Comparing our results with the findings from similar studies, it can be observed that our results are compatible with the vector biology and viral transmission cycle. Nevertheless, the influence of climate factors on the transmission of dengue depends on the regional location. Hence, our results implied that all the findings related to regional based climate factors are unique and local climate factors have a significant effect on dengue dynamics in CMC area. However, it is possible to modify the developed model to forecast the dengue outbreaks in given region by changing the real time data dependent parameter *n*(*t*).

During the modeling process of analyzing and forecasting tool, several limitation problems emerged. One of the main limitations for such model is lack of data sources. For an example, in the countries with relatively smaller geographical areas and commendable public transport services, the disease transmission dynamics is governed by human mobility. Hence, the forecasting model without human mobility does not have full capacity to capture the future outbreaks. However, the data availability is limited for the factors like human mobility. It would be purposeful to moderate the developed model to a spatial model by incorporating human mobility in further research [45, 49, 50].

Further, to analyze the realistic setting, factors like human behavior and awareness have to be added to the developed IR model. For instance, impact of the climate variability over disease transmission may be controlled by taking prior action such as cleaning the environment and destroying the vector breading sites. However, one of the major hindrance for such model is there is no proper way to measure the human behavior or socio–economic activities which affect the transmission of dengue. Changing the data dependent parameter including vector control policies will help to increase the accuracy of the model.

## Conclusion

Dengue is a disease with exponential growth. Since dengue transmission is being affected by climate changes, identifying the periodic pattern and correlation with climate conditions is vital to control the spreading of the disease. First, we considered the reduced classical SIR model and derived quasi–equilibrium IR model. Furthermore, we identified that there is no significant difference of qualitative and quantitative behaviour between reduced SIR model and quasi–equilibrium IR model. Then the data driven quasi–equilibrium IR model has been developed to capture the climate effect on dengue transmission.

Based on reported dengue incidences and climate data, we analyzed periodic and seasonal pattern of dengue infection for urban Colombo, Sri Lanka. Fourier spectrum over empirical data and precipitation data of CMC area have been indicated 26 weeks periodic pattern and it can be concluded periodic pattern depends on monsoon seasons. However, another Fourier amplitude related two and half year has been appeared. It would be worthwhile to examine the correlation between serotype shift and periodic pattern of dengue incidences in further research.

The correlation between dengue and precipitation has been analyzed and introduced the minimum and the maximum cutoff values to precipitation data. As a result we can conclude that if the weekly rainfall value for CMC area is in between 14*mm* to 454*mm* then dengue incidences have correlation with rainfall with 8-14 weeks lag. The correlation between dengue and temperature indicated the influence of ENSO.

Relating to analyzed data, the data driven quasi–equilibrium IR model has been moderated to predict weekly dengue incidences upto 4 weeks. Finally, the model has been validated and the model can be used to predict more than 75% accurate data. We are looking forward to extend this model using human mobility in further research.

## Data Availability

The data set used and analyzed during the current study is available from the corresponding author on reasonable request.

## Abbreviations

CMC: Colombo municipal council
SIR: Susceptible, infected and recovered
IR: Infected and recovered
ENSO: El Ni no southern oscillation
WHO: World Health Organization
